# Next Generation AT-Cut Quartz Crystal Sensing Devices

**DOI:** 10.3390/s110504474

**Published:** 2011-04-27

**Authors:** Vojko Matko

**Affiliations:** Faculty of Electrical Engineering and Computer Science, University of Maribor, Smetanova 17, 2000 Maribor, Slovenia; E-Mail: vojko.matko@uni-mb.si; Tel.: +386-2220-7111; Fax: +386-2220-7272

**Keywords:** quartz crystal, switching oscillating method, temperature characteristic compensation

## Abstract

Generally, AT-cut quartz crystals have a limited scope of use when it comes to high-precision measurement of very small impedance changes due to their nonlinear frequency-temperature characteristics in the range between 0 °C and 50 °C. The new method improving quartz oscillator frequency-temperature characteristic compensation is switching between two impedance loads. By modifying the oscillator circuit with two logic switches and two impedance loads, the oscillator can switch oscillation between two resonance frequencies. The difference in resonance frequencies compensates the frequency-temperature characteristics influence as well as the influence of offset and quartz crystal ageing. The experimental results show that the new approach using the switching method highly improves second-to-second frequency stability from ±0.125 Hz to ±0.00001 Hz and minute-to-minute frequency stability from 0.1 Hz to 0.0001 Hz, which makes the high-precision measurement of aF and fH changes possible.

## 1. Introduction

Quartz crystal temperature characteristics are of primary importance in high-precision measurement of small impedances. Generally, sensor techniques involve capacitance and inductance changes, particularly for the measurement of pico extensions, hollow pico-sphere magnetic properties, novel magnetic pico-adsorbents, displacement field forces, humidity sensors, Van der Waals force measurement, *etc.*

The application of quartz crystals for the measurement purposes using external electrical elements (*L*, *C*) that influence electrical equivalent circuit has been suggested in past articles dealing with capacitive and inductive measurements as well as with the use of two single quartz crystals [1–3]. Also described were the quartz crystal *C*_0_ capacitance compensation and high improvement of pulling sensitivity. The latter requires very stable frequency-temperature characteristics, determining the accuracy within a given measurement range. And even if an AT-cut quartz crystal with the angle of cut = 0 is selected, the temperature dependence of some ppm can still be registered [1–5].

Capacitive and inductive changes with the resolution up to 0.01 pF or 0.01 nH can also be measured with LCR instruments (Hewlett-Packard 4284A-Precision LCR meter, 20 Hz–1 MHz, 0.05%), however, once again the second-to-second and minute-to minute frequency stability plays an important role [6,7].

This research focuses on the temperature and ageing characteristics compensation of AT fundamental quartz crystals (5 MHz) operating over the measurement temperature range of 0 °C to 50 °C. Crystals fabricated in this manner exhibit excellent frequency *versus* temperature stability between 10 °C and 40 °C and also good start-up. For smaller frequency change measurement, however, the improvement of the frequency-temperature stability in this temperature range is of vital importance [8–10].

## 2. The Switching Method of Quartz Sensing Device

**Figure 1.**
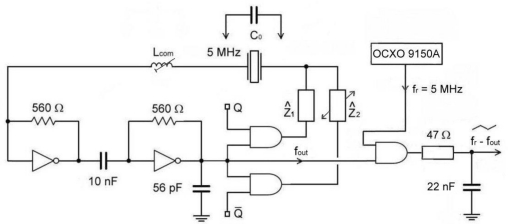
Quartz crystal switching oscillator.

The new measurement method is based on a quartz oscillator [11] and the switching part of the circuit, alternatively switching complex impendances *Ẑ*_1_ and *Ẑ*_2_ in the oscillator circle with the signal of ones and zeros *Q* and *Q̄*) representing a novelty in this research (Figure 1).

The output *f*_out_ represents the output oscillator frequency which is synchronously measured with regard to the switch *Q* and *Q̄*). The switch time duration *Q* is 1 s. When the complex impedances *Ẑ*_1_ and *Ẑ*_2_ are equal, then *f*_out_(*Q*) and *f_out_*(*Q̄*) are equal too. For this purpose, a special AT fundamental quartz crystal (cutting angle 0) operating near the antiresonance frequency 5 MHz has been selected. In the oscillator circuit, the inductance *L*_com_ is in series with the quartz crystal and together with the compensation method (*C*_0_) increases and linearilizes the frequency pulling range. The quartz crystal’s parasitic capacitance *C_o_* represents the capacitance of the crystal element and the holder [2]. Complex impedances are the same and can be capacitive (*1/jωC*) or inductive (*jωL*) in character. When the complex impedances are the same, *f*_out_ remains the same at *Q* and *Q̄* and depends on the quartz crystal resonant frequency *f*_0_, AT-cut quartz crystal temperature characteristics Δ*f* (*T*) and its ageing Δ*f* (*t*). However, when the complex impedances are different, the frequency *f*_out_ depends on the quartz crystal resonant frequency *f*_0_, the Δ*C*_2_ or Δ*L*_2_ change (frequency pulling) and AT-cut quartz crystal temperature characteristics Δ*f* (*T*) and its ageing Δ*f* (*t*). In case of the difference of both frequencies for *Q* and *Q̄*, Δ*f* (*T*) and Δ*f* (*t*) compensate because only one quartz characteristics is involved [9].

The ouput frequencies for both switching conditions are:

(1) $$f_{{\text{out}}_{1}}\;=\;f(Q)$$

(2) $$f_{{\text{out}}_{2}}\;=\;f(\bar{Q})$$

and can be expanded to:

(3) $$f(Q)=f_{0}+\Delta f(T)+\Delta f(t)+\Delta f(\text{counter}\;\text{error})$$

(4) $$f(\bar{Q})=f_{0}+\Delta f(T)+\Delta f(t)+\Delta f(\text{counter}\;\text{error})+\Delta f(\Delta C_{2})$$

When joining *f*_0_ and Δ*f* (Δ*C*_2_) (Equation 4), we get Equation 5 [1,2]. The particularity of this equation lies in the fact that it takes into account the compensation *C*_0_ and at the same time linearizes the quartz characteristics due to the Δ*C*_2_ change (Figure 1) [1,2,12].

(5) $$f(\bar{Q},k,\Delta C_{2})=\frac{1+\frac{C}{2\left(\frac{1}{k}C_{0}-\frac{1}{\omega_{0}^{2}\cdot k\cdot L_{com}-\frac{1}{\Delta C_{2}}}\right)}}{2\pi \cdot \sqrt{L\cdot C}}+\Delta f(T)+\Delta f(t)+\Delta f(\text{counter}\;error)$$

where:

*k* = 1, 2, 3: pulling sensitivity value [1,2],
*L* and *C*: mechanical behavior of the crystal element [1,2],
*L_com_*: compensation inductance,
*C_o_*: parasitic capacitance of the crystal element and holder,
*f*_0_: quartz crystal series resonant frequency.

(6) $$\omega_{0}=2\cdot \pi \cdot f_{0}$$

The pulling sensitivity in Equation 5 can be set with the value *k,* achieving at the same time simultaneous dependance linearilization Δ*f* (*C*_2)_ [1,2]. We get the frequency difference representing the temperature compensated and linear value of the frequency, which depends uniquely on the Δ*C*_2_ change. This means that it is dependent neither on the AT-cut quartz crystal temperature characteristics Δ*f* (*T*) nor its ageing Δ*f* (*t*) and nor the circuit temperature characteristics influences [Equations (7–9)] [1,2]:

(7) $$\Delta f(\Delta C_{2})=f(\bar{Q},k,\Delta C_{2})-f(Q)$$

(8) $$\Delta f(\Delta C_{2})=\frac{1+\frac{C}{2\left(\frac{1}{k}C_{0}-\frac{1}{\omega_{0}^{2}\cdot k\cdot L_{com}-\frac{1}{\Delta C_{2}}}\right)}}{2\pi \cdot \sqrt{L\cdot C}}-\frac{1}{2\pi \cdot \sqrt{L\cdot C}}$$

and:

(9) $$\Delta f(\Delta C_{2})=\frac{\frac{C}{2\left(\frac{1}{k}C_{0}-\frac{1}{\omega_{0}^{2}\cdot k\cdot L_{com}-\frac{1}{\Delta C_{2}}}\right)}}{2\pi \cdot \sqrt{L\cdot C}}$$

If *Ẑ*_2_ (Figure 1) is inductive in character, the equation would generally be similar with the inductance change Δ*L*_2_ [Equations (10) and (11)]:

(10) $$f(\bar{Q})=f_{0}+\Delta f(T)+\Delta f(t)+\Delta f(\text{counter}\;\text{error})+\Delta f(\Delta L_{2})$$

(11) $$\Delta f(\Delta L_{2})=f(\bar{Q})-f(Q)$$

While the specified counter accuracy (HM 8122) ±5 × 10^−7^ does not allow high precision measurements of small frequency changes at 5 MHz, the use of an additional reference frequency *f_r_* = 5 MHz (auxiliary OCXO oscillator) (Figure 1), of the frequency difference method (AND gate) and of the low pass filter enables very precise measurements of the frequency difference between the switches *Q* and *Q̄*. This output difference is defined with the Equation 12:

(12) $$(f_{r}-f_{Q})-(f_{r}-f_{\bar{Q}})=f_{\bar{Q}}-f_{Q}$$

The switching from *Q* to *Q̄* compensates the frequency *f_r_*, and consequently its frequency stability as well. The filter time constant for the frequency elimination is determined with the following equation:

(13) τ=R·C=47·22 nF=1.034 μs

## 3. AT-Cut Quartz Crystal Temperature Characteristics

**Figure 2.**
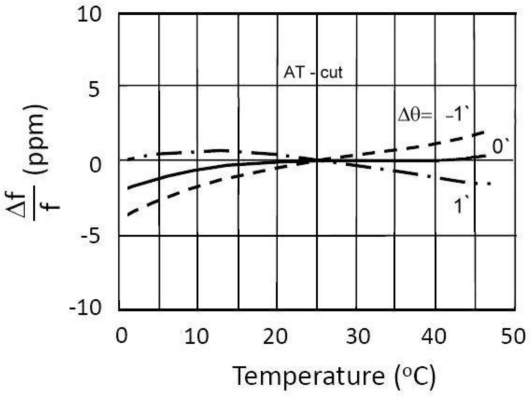
AT-cut quartz crystal temperature characteristics [12].

Due to their physical properties, AT-cut crystals are predominantly used in oscillator circuits. Their main advantage is the lower temperature sensitivity in the temperature range between 10 °C and 40 °C (Figure 2). The curves are represented as the cubical parabola with temperature intersection point lying between 25 °C and 35 °C, depending on the crystal cut angle and the mechanical construction. Equation 14 describes the crystal oscillation temperature frequency change (in ppm) with regard to the reference temperature [11–14]:

(14) $$\frac{\Delta f}{f}=A_{1}\cdot (T-T_{ref})+A_{2}\cdot {\left(T-T_{ref}\right)}^{2}+A_{3}{\left(T-T_{ref}\right)}^{3}$$

where:

*T*: environment temperature
*T*_ref_: reference temperature
*A*_1_ and *A*_3_: coefficients determined with regard to the angle of the cut

For higher accuracy, five measuring points (to measure the frequencies) or more may be necessary. By means of these, the best adapted cubical parabola is applied and the appropriate coefficients *A*_1_ and *A*_3_ determined. Nevertheless, this mathematical approximation is not precise enough for the high-precison measurements of small impedance changes [11–14].

## 4. Frequency Variation as Function of Time

**Figure 3.**
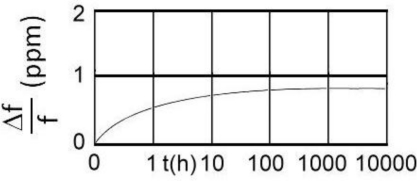
The quartz crystal frequency ageing [12].

Frequency variation is normally considered in short term stability (second-to-second and minute-to-minute temperature characteristics) and long term stability over days, months or years, called ageing. The short term stability of a quartz crystal depends on the actual oscillator design and is totally controlled by the quartz crystal at low drive levels (30 μW). The ageing rate is substantially influenced by the cleanliness of the resonator, the stability of the inert gas filling and the security of the final sealing process. Ageing is naturally greater during the first part of the life of the crystal unit. The frequency ageing can often be described by function of the form of time *t* (Figure 3) [11–14].

(15) Δf(t)=5·ln(0.5t+1)

It is necessary to distinguish between active and passive ageing. Active ageing is the frequency shift, when the crystal works under operating conditions—permanently oscillating in the circuit. Typically the ageing rates of the best cold weld crystals are less than ±1 ppm/year (10 °C to 40 °C). Passive ageing is the frequency shift during storage.

## 5. The Counter Error

Counter error Δ*f* (counter error) occurs in the measurement of the frequency *f*_out_ (*Q* and *Q̄*) (Figure 1). Typical counter (Programmable Counter/Timer HM 8122) accuracy is ±5 × 10^−7^ (through entire working temperature range +10 °C up to 40 °C), in 5 × 10^−9^/day after 48 hours continuous operation with crystal oven controlled (OCXO). Frequency repetition accuracy after 24 hours of “power off”: ±5 × 10^−8^. Resolution is determined as ±1 or 2 LSD, while frequency measurement accuracy is defined with the following term [6]:

(16) Accuracy:±(Resolution:FREQ+Time Base Uncertainty+Trigger Error: Measurement Time)

The novel switching method highly reduces the influence of the short- and long-term accuracy of the above described counter due to the compensation of previously mentioned influences of a single quartz crystal and the circuit as well as the influence of the difference method using additional reference frequency *f_r_* = 5 MHz. Frequency *f_r_* is produced by oven controlled crystal oscillator (OCXO 9150A) with a short-term stability (1 s) 5 × 10^−11^ (max) in the temperature range between 0 °C and 50 °C following the warm-up time of 30 min [15].

## 6. Experimental Results

The experimental data values in the 5 MHz quartz crystal equivalent circuit were measured by a HP 4194A impedance/gain-phase analyzer. The quartz crystal (HC-49/U) was selected due to its high Q value (Table 1) [14].

In Table 1, *f*_0_ represents the AT fundamental mode quartz crystal resonant frequency. *R* is series resistance and *Q*_q_ is quality factor [1,2].

For this research a quartz switching oscillator circuit (Figure 1) was experimentally selected switching between impedances *Ẑ*_1_ and *Ẑ*_2_ with the frequency 1 Hz. The impedances in this research are defined as *1/jωC*. The *C* values were in the range 2.5 pF to 40 pF. Within 2 s time, the counter measured both frequencies *f* (*Q*) and *f* (*Q̄*).

**Figure 4.**
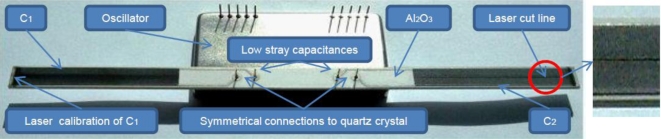
Oscillator circuit and capacitances *C*_1_ and *C*_2_ produced on *Al_2_O_3_*.

Two impedances *Ẑ*_1_ and *Ẑ*_2_ in the form of an open capacitor were used experimentally. The impedances were produced on a temperature stable material *Al_2_O_3_* and are of the same capacitance *C*_1_ = *C*_2_ = 4pF (Figure 4). Capacitances were produced by laser cutting and measure 5 mm × 40 mm in dimension.

**Figure 5.**
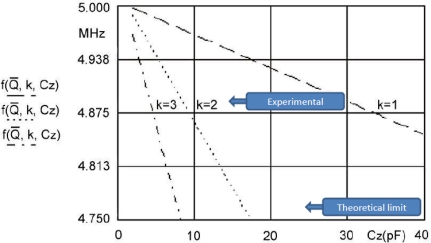
Quartz crystal sensitivity and linearity for *k* = 1, 2, 3 in the range *C*_z_ = 2.5 – 40 pF.

For three *k* values, three linearized characteristics and *C*_0_ compensation in the range of change *C*_2_ from 2.5 to 40 pF can be seen in Figure 5. The pulling sensitivity is highest at the value *k = 3*. For the temperature range between 0 °C and 50 °C (Figure 2) and *k = 3* the experimentally measured frequency change results (Programmable Counter/Timer HM 8122) were ±0.00001 Hz (second-to-second stability) and ±0.0001 Hz (minute-to-minute stability) representing 100 times better results than those achieved by existing methods in a given temperature range (in cases where the crystal is not extra temperature stabilized). At values *k = 1* and *k = 2* the stability is better, however the pulling sensitivity is lower.

## 7. Conclusions

Experimental results show that the use of the switching method excellently compensates AT-cut frequency-temperature characteristics and highly improves second-to-second and minute-to-minute accuracy by ×100 for *k* = 1, 2, 3, depending on *C*_0_ compensation. This high frequency difference accuracy represents a novelty and a major advantage of the switching method discussed in the measurement of ato and femto ranges. With fine tuning of a series load compensation inductance *L*_com_ connected in series with the crystal, the frequency of the oscillator is set to an appropriate output circuit frequency. It should also be emphasized that the exact pulling limits depend on the crystal’s Q value as well as the associated stray capacitances and the factor *k*. The inductance *L*_com_ is determined from known stray capacitances and the known factor *k*.

The factors affecting frequency stability such as wide operating temperature range, ageing and drive level as well as all other crystal characteristics influencing the stability should also be considered because a stable oscillator circuit plays an important role in the frequency pulling sensitivity increase. Frequency stability also depends on the temperature coefficient of the compensation inductance *L_com_* material. Stability of the electronic circuit depends upon the circuit type and quality of its elements. It is also important that the drive level of the quartz crystal does not exceed 30 μW [7,12,14,16]. These results clearly show that the switching method for the next generation AT-cut quartz crystal sensing devices outperforms conventional measurement methods.

## Figures and Tables

**Table 1.** Quartz data for resonant frequency 5 MHz [1,2,14].

***f_0_* (MHz)**	***R*(Ohm)**	***C*(fF)**	***L*(mH)**	***C****_o_***(pF)**	***L****_com_***(μH)**	***Q****_q_*
5	10	25	40.7	4	78.2	230153
